# 

**Published:** 1872-04

**Authors:** 


### INDEX.

### Vol. VIII to X Inclusive.

**Contributions.** PAGE.

About Homœpathy, P. H. Hayes, M. D ... 141
Affectation in Chronic Invalidism, P. H. Hayes, M. D ... 277
Character and Long Life, P, H. Hayes, M. D ... 250
Coming Exodus of Trade, Ben. H. Pratt ... 303
Doctors, Dr. Ned ... 114
Digestion, Exercise and Sleep, W. C. Coleman, M. D ... 253
Education before Birth, P. H. Hayes, M. D ... 5
Enjoying Poor Health, Ben. H. Pratt ... 165
Gifts and Gifts, Ben. H. Pratt ... 112
Hens and Health, T. K. Beecher ... 29
Hernia, D. L. D. Sheldon, M. D ... 33
High Toned Quackery, W. R. McLean, M. D ... 34
Healthfulness of Woolens, P. H. Hayes, M. D ... 305
Insanities of Sane People, P. H. Hayes, M. D ... 87
If I were a Doctor, Ben. H. Pratt ... 57
Inspectors, Examiners, Censors, Thos. K. Beecher ... 3
Nursing, B. B ... 143
Power of Imagination, P. H. Hayes, M. D ... 59
Powers and Properties of Water, P. H. Hayes, M. D ... 167
Profit and Loss, Ben. H. Pratt ... 275
Pat. Medicines, M. S. Bidwell, No. 1 ... 279
" " " " No. 2 ... 224
" " " " No. 3 ... 307
Personal Beauty, P. H. Hayes, M. D ... 221
Renewal of Life, P. H. Hayes, M. D ... 113
Some Obsolete Aphorisms, Ben. H. Pratt ... 139
Surgery, Esthetics of, Ben. H, Pratt ... 193
Summer Resorts, Ben. H, Pratt ... 247
Things Sanitary, Ben. H. Pratt ... 32
Tough, Tougher, Toughest, Dr. Ned ... 6
Vital Relations, P. H. Hayes, M. D ... 195
Women's Rights, Ben. H. Pratt ... 85
Wine and Brandy, P. H. Hayes, M. D ... 20
Wholesome Heat for Healthful Homes, Ben. H. Pratt ... 1
Woman Question, D. L. D. Sheldon, M. D ... 116
Work Day, Play Day, Ben. H. Pratt ... 219

**Clippings.**

Antipathy of the sexes ... 241
Brain wasting ... 110
Betel nut ... 144
Colored Deacon's Prayer ... 9
Doctor's Difficulty ... 110
Deaths at 5 A. M ... 9
Dyspepsia ... 218
English Groom and Yankee ... 214

PAGE.

Effects of tight lacing ... 11
Experiments on Frogs ... 134
Food for Infants ... 72
How to Soften Hard Putty ... 100
Hygenic Rules ... 110
How long to Starve ... 82
How to Sleep ... 134
Illinois Doctor ... 61
Ingenious Wife ... 72
Influence of marriage upon Health ... 308
Japanese Domestic Life ... 172
Jewish Physicians in Rome ... 241
Lines to a Skeleton--Poetry ... 91
Medical Preaching ... 116
Odor of Plants ... 110
Promotion of Anatomical Science ... 17
Pulse of Animals ... 204
Save the Babies ... 105
Sick Headache ... 169
Sand Club ... 197
Scotch Humor ... 225
Too Little Sleep ... 69
Transplanting Hair ... 144
Tea Drunkards ... 225
Vine culture in France ... 182
Weather, to Predict ... 225

**Correspondence.**

Letters exposing Quacks and Humbugs, with other communications of interest to the people, 15 41-67-96-125-151-176-205 233-262-287-316.

**Household Hints & Recipes.**

Containing valuable hints for ordinary ailments, together with Recipes of use in every household.

Alabaster, to clean ... 70
Burns ... 72-127
Bleaching Compound ... 45
Bowel Complaint ... 45
Bruises ... 17
Bleeding, to stop ... 17
Beer, Root, to make ... 157
" Ginger, to make ... 266
Baldness ... 179-265 235
Bread, to preserve ... 208
Boots and Shoes, breaking in ... 209
Breakfast Dish ... 290-291
Beef Tea, to make ... 292
Balky Horse to cure ... 264
Cement ... 44-98-236-318
do for Acquaria ... 288
do for Marble ... 318
do for Metals ... 318
do for Glass ... 318
do for Stoves ... 128
Colored Chalk ... 98
Chronic Diarrhœa ... 98
Coal, to save ... 98
Clothes Pins, to save ... 70
Castor Oil, palatable ... 17-70
Cologne ... 71-180
Cushions, air ... 71
Chapped Lips ... 72
Cracked Wheat ... 45
Cough Mixture ... 17
Chilblains ... 18
Chafes ... 18-179
Cream, substitute for ... 18
Colds ... 129

PAGE.

Colds in the head ... 18
Cleaning Stoves ... 127
do Kid Gloves ... 154
do Paint ... 71
do Gilt Jewelry ... 156
do Gold Chains ... 44-179
do Looking Glasses ... 210
do Straw Hats ... 18
do Bottles ... 256
do Silver or Gold Lace ... 71
do Brass ... 71
do Straw Matting ... 17
do Alabaster ... 70
do Floor Cloths ... 71
do Gilding ... 127
do Silver ... 18
Celery as a nervine ... 128
Corns ... 128-289
Cordial, Blackberry ... 179
Carpeted Floors ... 208
Catsup ... 289
Chicken Cheese ... 291
Coffee and Milk ... 282
Consumption ... 292
Colic in a filly ... 264
Counterfeits, to tell ... 265
Carmine, to render brilliant ... 317
Disinfectant ... 100
Dysentery ... 18-265
Diarrhœa ... 98
Diabetes ... 127
Diabetic Bread ... 235
Depilatory ... 290
Dandruff ... 264-290
Distemper ... 264
Drawings, to protect ... 235
Eating when sick ... 236
Eraser for grease ... 236
Fruit in Rooms ... 100
do to keep ... 70
Flowers, to preserve ... 45
do to crystalize ... 156
Flies, to kill ... 17-264
do to keep from horses ... 154-236
Fly paper ... 266
Furniture, to restore ... 18
Fever, cold ablutions in ... 157
Fried Bread ... 207
Fish, as a diet ... 210
Fetid Feet ... 264
Fuel ... 236
Grease, to remove ... 70
do Tar and Pitch, to remove ... 290
Gilding, to improve ... 127
Gum-Arabic ... 181
Gold Powder ... 181
Glue, ether ... 207-266
do liquid ... 155-318
Garlic, to remove from milk ... 264
Grapes, to preserve ... 319
Hams, to cure ... 72-99
Headache, turpentine in ... 208
do congestive ... 70
Horseradish ... 155
Honey, tomato ... 179
Hair dressing ... 180
do Wash ... 208
do Tonic ... 210
do Curling ... 289
Horses, overheated ... 264

PAGE.
Hay Asthma....235
Handles, to fasten....317
Hardening small tools....317
Itch....18
Ink, indelible....18
do do red....290
do copying....264
do marking, crimson....289
do to remove....154-155
do Green....319
do Blue....319
do Yellow....319
do Red....319
do Gold....319
do Silver....319
do Black....319
do White....319
Ingrowing Nails....208
Iron Rust, to remove....18-289
do Taste, to remove....291
Iced Apples....264
Jam, preserving....304
do raspberry....154
do blackberry....154
Jelly, of meat....318
Kalsomining Fluid....265
Leaky Roofs....98
Lemons, use of....72
Labels for bottles....128
Liquid Glue....155-318
Lice on cattle....265
Mucilage....155
Mirrors, to repair....156
Moth Preventive....207-209
Mushroom Catsup....288
Milk or cream, substitute for....289
Mock Venison, of corn beef....290
Mice, to keep away....264
Mocking Bird Food....264
Marking Ink, crimson....289
Offensive Breath....71
Onions....47
Odor of Flowers, to collect....156
Oiling Harness....180
Pills, to swallow....181
Pudding....208
Poison from Orange peel....210
do do Confectionery....99
do do Hair Restorers....44
do do Ivy....18-45
do do Insects....154
do do Colors....46
do What to do in....45
Preservation of Essence of lemon....288
Pumpkin Preserves....229
Pickling Green Corn....910
Posts, to preserve....264
Paste, perpetual....235
do for bottle labels....181
Polishing Stoves....317
do Silver....44
Plants, unhealthy....99
do in winter....100
Putty, to soften....100-318
do and paint, to remove....154
do French....180
Potatoes, to keep....71
Potato Puffs....154
Papering Rooms....47
Paint, to remove....17
Piles....180
Polish, for Patent Leather....181
Quack Bitters....288
Rust, to prevent....71
Rawhide, use of....209
Rubber, substitute for....210
Roup remedy....266
Shoe Blacking....290
Scratched Furniture, to restore....292
"Scratches" in Horses....127-264
Sauce, Worcestershire....265
Stammering....266
Sleep and Sunshine....236
Starch, improved....318
Stain for wood....98 319
Sealing Wax....70
Scarlet Fever....70
Silver or Gold Lace, to clean....71
Shaving Fluid....46-289
Sore Throat....46
Sick Room, noise in....46
Summer Suits, to wash....47
Straw Matting, to brighten....71
Silver, to clean....18
Straw Hats, to whiten....18
Spectacles, how to use....178
Sweeping Carpets....155
Soap....179
Sick Stomach....207
Salad Sauce....208
do Dressing....289
Scarlet Fever, Croup, &c....208
Sponges, to bleach....288
Soft Cheese....289
Tomatoes, to keep....70
do catsup....70
Throat Diseases....38
Tooth Ache....18-209
Tar, to remove....128
Tobacco chewers....154
The Teeth....154
Tea, uses of....180-210
Tallow, to purify....207
Toilet Vinegar....210
Trap for Insects....291
Tanning Leather....291
Thumb Sucking....235
Varnish, to imitate ground glass....98
do for ladies' shoes....98
do for photographs....71
do waterproof....155
do gold, for picture frames....181
do flexible....264
Verbena Water....98
Ventilation....319
Washing hands with turpentine....98
White Wash....70-180
Wrought Nails, cheap....44
Whooping Cough....128
Waterproof Compound....154-156-235
Winter Salad....155
Writing, to restore....155
Wax, for flowers....179
Wine, blackberry....179
Washing Compound....181
Whitening Smoked Walls....181
Water for Houses....207
Weights....207
Welsh Rarebit....209
Washes, colored, for out-door....291
Why a child loves sugar....292
Wasp Stings....264
Weeds, to destroy....235

**Medical Items and News.**

Containing the latest and most reliable Formulæ for the treatment of all manner of Disease. Culled from the Standard Medical Journals of the world; carefully arranged by the Editor.

Asthma....10-91-119
do spasmodic....198
do cigarettes for....282
Antidote for Carbolic Acid....35
do do Strychnia....119
do do Arsenic....256
do do Phrenic Acid....280
do do Opium....170
Albuminuria....10
Anasarca....11
Aperient and Alterative for children....120
Anatomical Specimens....120
Anthrax....122
Antibillious Pills....123
Adhesive Plaster....226
do do to remove....171
Abscess....198
Acetate of Lead Lotion....282
Ascaridies....257
Angina Pectoris....227-230-259
Aromatic candles....228
Aspiration, in hydrarthrosis of knee....230
Anodyne....309
Bleached Tincture of Iodine....92
Beriberi....93
Biliary Calculi....63
Buboe....64
Bronchitis....65-311
do chronic....121-226-283-310
Burns....10-18-150-170-175-257
Baker Brown....117
Boils and Whitloe....118-145
Bright's Disease....145-170-227
Bronchitis....148
Bellevue Hospital....150
Baldness....203
Bichloride of Methylene....281
Bowels, pain in....281
Bromism, symptoms of....309
Coryza....256-259
Cough Syrup....38-256
do Mixture....120-226-258-282
Consumption, climate in....260
Caoutchouc....228
Cystitis, chronic....310
Catarrh and colds....122
Catarrh of Bladder....311
Chest Affections....315
Cod Liver Oil....286
do do do Emulsion....92
do do do Tasteless....257
Chapped Hands and Lips....93
Constipation....94-117-171-171
Chilblains....94-201-256-310
Cholera....10-63-172-256-257
do Infantum....96
do Morbus....282
Cerebro-Spinal-Meningitis...61-64-118-146-281
Carminitive Powder, for infants....62
Castor Oil, to disguise....63
Chloroform, administration of....63
Carbuncles....65-286
Cyanosis....66
Chloroform and Glycerine....36
Cathartic Pills....36
Croup....37
Calculus, biliary....37-173
Chloride of Potassium....37
Cancer....40
Chloral, cases treated with....40
Canned Fruits....45
Carbolized Glycerine....9
Copavia....11
Colodian....121
Cirrhosis and Albumniuria....122
Caffeine....122
Colds....123
Carbolic Acid as a local anæsthesia....145
Chloral do do 281
Chapping of the skin....170
Croup....172
Chancroids....198
Chloral with Cod Liver Oil....199
Creosote, to give....282
Colic....283
Diaphoretic....64-283
Dyspepsia....9-65-123
Dissecting Wounds....36
Delirium Tremens...38-40-175-198-233-312
Diabetes....10-202-258-280
Diphtheria....11-280-282
Dysentery....12-283
Dissections in Canada....117
Drs. Baker and Thomas....117
Declined....117
Dropsy....145
Diarrhœa, chronic....171-309
do in teething....147
do infantile....201
do of phthisis....313
Diabetes....170-258
do mellitus....149
Drop Wrist....150
Depilatory Mixture....256
Epilepsy....10-12-13-92-199
Epileptic vertigo....13
Eczema....62-117-145-146-171-226
do capitis....200-312
do papulosum....309
Emmenagogues....63
Epithelioma....66
do of eye....226
Encysted Tumors....37
Ear, diseases of....14
Expectorant Mixture...28 120-256-226-358-282
Erethema....145
Erysipelas...145-151-170-173-174-199-227-229-260-309-313
Exophthalmic Goitre....146
Ether, inflammability of....199
do administration of....226
Empyema....202
Emulsion of Raw Beef....285
Epistaxis....229
Ergot....310
Felons....37

PAGE.
Fever, Malarial ... 11
do Intermittent ... 12-174-203-282
do Yellow ... 117
do Typhoid ... 171
do Infantile ... 146
do treatment of ... 173
Freckles ... 119-280-280
Fissure of Nipple ... 149
Fetid Feet ... 264-282
Flatus ... 257
Gonorrhœa ... 9-171-257-258
do injections for ... 93
Glycerine of Tar ... 61
do Ointment ... 118
do Jelly ... 311
Goitre ... 11-64-172
Gleet ... 36-258
Glanders, in man ... 10
Gastralgia ... 118-259
Gelseminum ... 123
Gastro-enterites ... 198
Granular Lids ... 200
Gout ... 201
Garlic, compound syrup of ... 280
Hemorrhage in Typhoid Fever ... 92
do Internal ... 313
Heart, disease of ... 202
do irregular ... 226
do irritable ... 284
Heartburn ... 92
Hemicrania ... 37
Hydrocele ... 38-226
Hemorrhoids ... 281-310
Hemorrhoidal Suppository ... 118
Hops, as an external application ... 119
Hay Fever ... 119-315
Hæmoptysis ... 147
Hemi-chorea ... 200
Hydrophobia ... 200
Headache, after drunkenness ... 280
Hair Tonic ... 210-282
Hoarseness, chronic ... 226
Hydatids ... 311
Hydrate of Chloral ... 315
Incontinence of Urine ... 256
do do in the aged ... 9-280
Incontinence of Urine in children ... 35-91
Iodiform ... 61
Impotency ... 36
Influenza ... 36
Ingrowing Toe-nails ... 118
Iodine tincture, bleached ... 171
do to deprive of stain ... 284
do caustic ... 280
Insanity ... 173
Itch ... 283
Jallap Biscuits ... 281
Laxative Mixture ... 91
do Pill ... 92
do Powder ... 145
Lacto-phos-Lime ... 57
Lostorfer's Corpuscles ... 38
Liquorice, compound powder of ... 40
Lip Salve ... 120
Local Anæsthesia ... 121
Lice ... 121
Lipoma, diagnosis of ... 172
Loss of Blood ... 203
Liniment ... 283
Liquor Sedativus ... 283
Litchen, Syphilitic ... 313
Mouth Wash ... 118-257
Malignant Pustule ... 173
Menorrhagia ... 281
Night Sweats ... 170-175-310
do do in Phthisis ... 91-227-256-313
Nævus Maternus ... 62
Neuralgia ... 120-147-148-256-314
do uterine ... 282
do hip and thigh ... 282
do facial ... 284
do in infants ... 229
do snuff ... 170
Nose, removing foreign bodies from ... 121
Nit. of Amyl ... 150
Nervous Sedative ... 204
Nocturnal Pains ... 227
Nipple, Fissure of ... 149
Otitis ... 10-61
Opium Habit ... 38
do Antidote ... 170

PAGE.
Opium Cure ... 259
Os Uteri, rigidity of ... 410
Odontalagia ... 12
Phthisis ... 150-198-231-314
Prepared Earth ... 151
Pleurisy ... 62
do subacute ... 170
do with effusion ... 313
Perinœrrhaphy ... 170
Pruriginous Affections ... 171
Phosphorus Mixture ... 198
do Etherial Tincture of ... 256-309
Perchloride of Iron ... 200
Pulse of Animals ... 204
Purgative ... 280
Puerperal Convulsions ... 281
Prolapsus Ani ... 259
Pulmonary Cavities ... 261
do Hemorrhage ... 119
Pepsine and Pancreatic Emulsion ... 228
Post Partum Hæmorrhage ... 93
Pruritus ... 93-147-284
do Vulvæ ... 37-62-311
Peritonitis ... 35
do Pelvic ... 94
Plaster-of-Paris Splints, removal of ... 61
Psoas Abscess ... 63
Pertussus ... 64-147-199-311
do signs of ... 309
Pneumonia ... 66-146-175-227-280-280-312
Psoriasis ... 38
Poisoning by Strychnia ... 9-119
do do Opium ... 9
do do Rattlesnake ... 13
do do Carbolic Acid ... 35
do do Phrenic Acid ... 280
do do Arsenic ... 256
do From Orange Peel ... 210
do do Confectionery ... 99
do do Hair Restorers ... 44
do do Ivy ... 18-45
do do Insects ... 154
do do Colors ... 46
do What to do in ... 45
Pure Air, Test for ... 118
Perspiration of hands and feet ... 118
Post Mortem Napoleon III ... 124
Piles, internal ... 148
Rheumatism ... 62-149-151-176-199-226-228-258-280-282-283-285
Rubiola ... 39
Retention of Urine ... 39
Revaccination ... 14
Ringworm ... 145-227
Rabies ... 312
Spina Bifida ... 92
Syrup of Lactophosphate of Lime ... 93
do Cubebs ... 120
do Borax ... 285
do Morphia, Comp ... 256
Syphilis ... 93-281
Syphilitic Ulcers ... 95
do Skin Disease ... 174
Small Pox ... 36-63-94-118-122-124
Sick Headache ... 62
Suppository ... 10-62
do of Chloral ... 309
Sloughing Ulcers ... 35
Suppuration ... 35
Scabies ... 12
Strychnia, Hypodermically ... 12
Singular Accident ... 13
Spermatorrhœa ... 14-199
Spasm of Glottis ... 118
Styptic ... 122
do Cotton ... 119
Strumous Ophthalmia ... 121
Summary Therapeutics ... 123
Sick Headache—Nervous ... 145
Sciatica ... 145
Sweating ... 146-198
do from Phthisis ... 232
Sprains ... 151-175
Scarlatina ... 175
Surgical Dressings ... 175
Subcutaneous Injections ... 198
Subcutaneous Injections in the year 1664 ... 228
Skin Grafting ... 201
Seborrhea ... 284
Spinal Anæmia ... 261

PAGE.
Synovitis, chronic ... 226
Strumous, children ... 228
Surgical Dressing ... 229
Suppression of Milk ... 231
Show Bottles—liquid for ... 312
Tetanus ... 62-200
do Traumatic ... 9-35-91
Tic-doloureux ... 61
Tape Worm, 11-12-36-62-150-233-258-285.
Tumors, Encysted ... 11
Typhoid Fever ... 171
Test for Albumen ... 200
Test for Saccahrine Urine ... 227
Toothache Drops ... 258-280-310
Tonic Prescription ... 310
Uterus, Amputation of ... 10
Uterine, Neuralgia ... 282
U. S. Dispensatory ... 117
Uvula, Elongation of ... 145
Ulcers ... 170-198-236-257
do Varicose ... 148
do Indolent ... 172
do Syphilitic ... 95
do Sloughing ... 35
do Venerial ... 39
Vaccination, substitute for ... 94
Varix ... 95
Vomiting ... 36
do of Pregnancy ... 36-262
do after meals ... 262
do from cough ... 284
Veratrum Viride, Externally ... 146
Vienna Exhibition ... 149
Whooping cough ... 64-199-316
Wens ... 31
Witness necessary ... 117
Whitlow ... 145
Xyol ... 39
do in Small-pox ... 124

**Editorial.**

Astigmatism ... 21
Abuse of the Eyes ... 48
Baby ... 212
Babies, Their care ... 77
Club Foot ... 187
Close of Volume ... 211
Catarrh ... 270
Cleft Palate ... 320
Cornea ... 321
Deformed Feet ... 322
Eye cups ... 23
Elmira Surgical Institute ... 182
Eye Lids ... 239
Fussy ... 103
Felon ... 20
Heating Apparatus ... 131
House Drains ... 134
Health Journals ... 158
Healthy Dwelling Houses ... 296
How We Spent Our Vacation ... 267
Hot Weather ... 271
Is It Murder ... 22
Keep cool ... 158
Knock Knee ... 293
Late ... 129
Medicine and Doctors ... 73
Magnetic Waters ... 74
Morbus Coxarius ... 268
Muscæ Volitantes ... 269
Ninth Year ... 129
Near and Long Sightedness ... 294
New Postal Law ... 320
Our Premiums ... 73
Our Joints ... 213
Ophthalmoscope ... 240
Pterygium ... 130
Purulent Ophthalmia ... 185
Prevailing Excitement ... 237
Queer Remedies ... 102
Reply ... 102
Reading After Confinement ... 296
Read It ... 297
Strabismus ... 75
Sea Sickness ... 50
Spectacles ... 159
Spinal Curvature ... 182
Staphyloma ... 211
Time ... 101
Tattooing ... 101
Tear Passages ... 104
Tobacco, Use of ... 77
To the Point ... 19

PAGE.
The Code....137
That Law....293
Watering Places.... 49
Wry Neck....211
Yardly Swindle.... 19

**Editorial Chit-Chat.**

Comments upon occurrences of the Day.

At it Again....106
A New Vein....106
A Blessing.... 107
Academy of Sciences....79-107
Artificial Eyes....108
A Horse Device....108
Attention, Sir Knights.... 78
Aernautic.... 81
Announcement.... 52
After Skulls....135
A New Artist....135
Artemus Ward....163
Ambulance....189
Annoyance....299
All Aboard....271
A Shot....271
A Bull....243
Another....243
Advertiser, The Daily,....244
Busy.... 79
Bridge Piers.... 81
Babies.... 51
Balloon.... 51
Burial, New Means of.... 53
Back Numbers.... 25
Business....135
Barry Gray....135
Balloon Voyage—The Graphic....164
Busy.... 189
Ballooning—Prof. King....189
Baby Farming....190
Blessing after all—the Potato Bugs 297
Beautiful Roe—Burnt district....271
Coon Chase, at Scranton....106
Cripple Manufactory....106
Cockroaches....109
Charles Palmer.... 79
Conderango.... 80
Cheap Advice.... 81
Cozy.... 82
Cerebro-Spinal-Meningitis.... 51
Cows.... 27
Central Park Museum....135
Centennial....136-324
Cranford....162
Chandler....162
Centenarian....217
County Fair....297
Cataract....271
Cremation....272
Corns....236
Clever of Them....243
Dangerous....107
Death of an old Friend.... 80
Don't Vote Right.... 25
Discontinued....135
Dixey....162
Declines....162
Does Well....162
Don't, Please....190
Disgraceful....190
Don't....297
Did Not Pay....299
Dental Bill....299
Dusting....300
Delay....244
Dexter's Card....244
Doctor Luke....324
Exasperating....106
Elegant....107
Egotistical Ass—T. Curtis Smith.. 53
Exquisite.... 25
Experts.... 26
Envelope....217

PAGE.
Emotional Insanity....243
Frasier House....26-79
Fine Printing.... 80
Famous.... 81
French Diet.... 82
Fitness of Things....189
Found at Last....272
Fashion.... 243
Fastidious, Very....243
Gas, Cost of....108
Gum, Chewing.... 51
Greeley, Horace.... 52
Grace, in Philadelphia....137
Gregg, Dr....162
Granger....298
Give it up—a conundrum.... 271
Good....243
Gazette, Daily....271
Halls....106
Hot.... 79
Heating New School....136
Hard Up....137
Hairy Family....137
"H"—Jokes From....137-189-216-298
Ivy Poison.... 79
Iron Prints.... 80
Inebriate Asylum.... 51
Indianapolis.... 53
Imagine His Feelings—H's Joke.137
In the Nick o' Time....217
Inconvenience of Deafness....298
Insane....243
Index.... III
Johnson, Dr....162
Japanese Medical Journals....299
Just Think of It....299
Liquor Dealers....298
Large Salary....106
Lectures....80-107
Lunatic Asylum.... 53
Lady Physicians....162
Law and Medicine....243
Musical Insurance....106
Mad Doctor.... 82
Medical Men.... 26
Microscope....135
Mayor Caldwell....135
Masonic Temple....190
Malt Extract....190
Meat Question....298
Medical Items and News....272
None, If You Please....108
Nealaton, Dr.... 78
New Medical Society....272
No Go.... 79
Noncommittal.... 79
Novel Fishing....26
New Citizens.... 80
New York.... 80
New Order In Masonry.... 78
Newspaper Law.... 26
New Insurance....135
New Dress....135
New Cure....136
New Bridge....189
New Tenor....217
New Physician....297
Needs Protection....300
Note It....272
Old Boots, Use For....107
Our Contributors.... 78
Ourself.... 54
"101"....135
Old Times....136
Our Schools....163-272
Our New Place....214
"Once A Month."....216
Old Subjects....297
Olden Time Surgery....297
Our Barometer....300
Only a Flower....324
Profitable Sutling....106
Prof. Ford.... 79
Prof. King.... 79

PAGE.
Professional Ladies.... 89
Photographic Invention.... 70
Personal....25-51-51-81-162
Pleasant Valley Wine Co.... 52
Prudent....136
Passed....163
Park....190
Proprietory Medicines....191
Penn. vs. N. Y....216
Precaution....217
Probable....243
Prayed Out of Town....244
Patent Medicine Proprietors....244
Plate Glass....324
Quack Medicines....298
Queer....272
Queer Medicines.... 245
Rare Binding.... 79
Rascally.... 52
Rough on a Hartford Physician..136
River Bank....156
Rough on Dr. Johnson of Chicago.243
Roller Towels, Danger of....243
Railway Accidents....244
Rest....245
Soup Fountains....106
Solid....106
Sea Sickness....107
Simple and Reliable....107
Spelling....108
Strong Firm.... 78
Shooting.... 79
Smillie, Artist.... 79
Scranton.... 79
Sharp Rogues.... 80
Sleep, in Infants.... 52
Siamese Twins—to Make....135
Social Club....135
Social Evil Hospitals....136
Scientific Lectures....137
Scranton Times....162
Strawberries....162
Street Railway....162
Sabbath, How to Spend....163
Surprised, But Not Defeated....164
Swindling....190
Sad Death....190
Spain....216
Swallowing a Tool-Chest....297
Splendid Organ....299
Surgical Novelties....272
Severe Struggle....243
The Envelope....106
Tea, Rival of....106
Too Thin....107
The Artists.... 79
Tyndall.... 79
The Sun.... 81
Tape Worm Mania.... 82
Too Pleasant by far.... 26
Troublesome....162
That Infernal Noise....298
Try It....300
Tapeworm, in Grouse....300
Thunder Storm....272
Turtle Club....273
The Boys....273
Too Bad....243
Twins....244
Ten Years for Bistoury....244
Tobacco Tax....244
The Woman....244
Unique Device.... 26
Warning....106
Which?....216
Wines, American....216
Welsh....217
Wanted.... 297
Why Not?....298
Whew! Cost of Chinese Servants.301
Why Is It? To Tell a Married Man....271
Would Not Set—Our Robin....273
Wonderful Lake....324

### SUBSCRIPTIONS RECEIVED.

In this column we will credit every subscription received during the last quarter. Subscribers will please notify us immediately if their payments are not duly credited. The state only is given, this, with the full name of the party subscribing, will be sufficient to indicate who is meant:—

NEW YORK.—Dr G W Levitt—W E Slocum—Mrs R Smith—T C Cowen—F D Ramsdell — Dr B A Fordyce—Merwin Buckley—W H Wilson—Dr M L Burbank—Dr H D Wells —William Doderer —Wm Irish — S H Ainsworth—Peter Vanderwater—Dr Seely—S G Ellis — Dr E G Derby—Mrs I Caldwell— Dr J C Dixon—N Hawks—F M Jones—J H Marshal— D L Holden—O Groom—C W Gardner—H B Smith—W H Longstreet—W E Hart—H J Gilbert—S H Wadsworth—Mrs Preswick—Dr Sayles—M T Millius—W M Farrar—S E Brace—Dr W L Appley—H W Norman—John Callist—Dr S A Cottrell—F G Hall —Dr J Everitt— Dr P M Barclay —W O Thayer—Mrs L M Keeney—Dr Wm Coryell—Mrs I N Johnson—James Carpenter—Dr H R Rogers—John Griswold—C Breed—Dr B W Dewey–S C Blauvelt—Peter Boner—Rev Wm Atwood — A B Abbot — B Kadder — Capt C Ulman—G W Gould—E C Hopper—S R Page—James Chapman—C V Bush—P S Smith—M J Ellis—Dr H D Seaman--J Luce —J T Beach—L Halstead—N Sherwood —W Ford—W O Obour—J Clough—C M Greatsinger—Mrs M Lane—Frank M Reed—H B Gilbert—Mrs Amos Fuller—Mrs J Adams —Dr S B Sellen—F D Brown—M T Babcock—J Beardsley—Mrs C L Fitch—W F Cooper—Wm Johnson—Mrs J Stanwood—Mrs D Buck--S W Adamy—Dr E S Smith—Thos Porter—W S Cole—Dr J M Hagiy—M J Jourdon—Prof B G Wilder—J D Williams—Dr H N Burr—G A Gerrow—Dr W Booth—Dr C Mudge--L C Taber—A N Wood—H Burden--L Clarke—N Bence--E Vines—Frank Everets—L Sherrill—J Mosey—G Kolfice—Kate L Clark —Mrs Jane Greene—S Sturgen—A L Heseltine—Mrs Jos Harper—Mrs J H Hayt—Mrs E R Parker—P Clark—Jesse Owen.

PENNSYLVANIA.—Dr G B Curtis--M W Cole--Dr N W Stroup--L R Bender--D C Scoville--J A Gamage--L F Clark--Jas A Brown--L L McCall -- C C Finch--Dr C A Havens--I W Mead--N A Monroe--L French -- G W Barney--P L Pettengill--S S Packard--G W Hoover--W P Scarf--Dr S W Dayton--S R Stibgen--Dr A R McCarthy--H W Kalisch--L W Cushing--Geo Bogle--J C Wollison--J T Parke--D C Hendy--H W Williams--D Ramback--J E Lloyd -- Prof A S Low -- C F Schaffle --W A Wallace--Rev R Smith--Dr W C Coleman--Noah Leonard--M B Weaver--Alexander Reede --J M Fletcher --Dr M D L Dodson--H C Eyer--E G Martin--W W Irvine --Dr A Chamberlain--W C Hyde--U Updegraff--H Shaw —Miss E D Sweely—Dr J F Kocher—Thos Wright—D W Kelley—M S Wilt—Dr T Bougger—P Hillbish—Ida Bliss —W F Eckbert — Kate Jessup—Dr N W Stroup — H H Coe—H L Holden—D W Smith—T D Hudson—C S Sellard —D Martin—Mrs H Beard—Mrs E Stratton—Dr A S Cummings—J C Shaeffer—J H Whitney—Jas Bowman—Dr J Y Shindel—J B Gilson—Mrs J E Mitchell—E T Tremain —Dr M L Davis—Dr M L How—Dr Alex Craig—N Shoemaker— Dr W N Miller—A G Walter — Dr S T Davis—Miss Julia Petitt—Dr A S Lovett—Dr W U Truckenmiller—Dr W W Cable—A A Root—Dr I D Hopkins—John Shippen—Drs A Harshburger & Son.

ALABAMA.—Dr J O Zeigler—Dr W S Forwood—Dr B F Coleman —W E Johnson —A H DeYampert — Dr J Huggins—Dr J M Collier.

ARKANSAS.—Drs Hooper & Breysacher — Dr C B Knode.

CONNECTICUT.—E Beach.

COLORADO TER.—P R Thombs.

DIST. COL.—H Cushing--C E Coon.

FLORIDA.—C B Wilder—E T Tabal—Dr A J Wakefield—Dr Geo A Penny.

ILLINOIS.—Dr T. J. Whitten—Dr W Bradley—Dr W H Crane—Miss Eliza Griswold—Dr R McLareu.

INDIANA.—Dr Soutnwick--Mrs Flora Southwick--A D Kimball--Dr J S Shively--Dr A P Morray—John Dury--Dr J L Wooden--M H Weir--Drs Kemper & Boyden.

KANSAS.—Dr G D Mason.

KENTUCKY.—Robt A Herring—J S Shepard.

MINNESOTA.—J C Euster.

MAINE.—B L Crooker—E P Snow.

MASSACHUSETTS.—E F Hall — J M Dunell — F G Welch—Dr S W Hayes—C F Brownell—Dr W S Brown—Mrs A McGrath.

MONTANA TER.—A C Hall.

MISSOURI.—Mrs W A Tubbs—Jas McDonald.

MICHIGAN.—Dr H. Loomis—Mrs Cooper— J W Mandigo—Mrs D C Richards—Dr L R Dibble—H S Tyler—Dr T F Dodge—Miss A M Hurd—Dr Cooper — Dr DuBois—Dr Yost—A T Watson.

MISSISSIPPI.—Dr J G Montgomery.

NEW HAMPSHIRE.—Edward Bunnells.

NEBRASKA.—Dr S L F Ward—Dr J Cochran — Chas W Key.

NORTH CAROLINA.—Dr J C Leathers.

OREGON.—Hiram Smith.

OHIO.—Mrs O Williams—M Webber-Dr W M Chenesy.

TENNESEE.—A A Davidson—Thos Lattimer—Dr F R Bailey.

TEXAS.—Dr Jno Hoffman.

VERMONT.—Dr L D Ross.

WISCONSIN.—B B Borden--Mrs M P Crawath—W H H Gray.

IDAHO TER.--J E Stuart.

NO ADDRESS.--Henry Minere--Mrs Chas R Hemingway.

NO NAME.--Jacksonville, Fla.

### THE BISTOURY GIVEN AWAY.

OUR TERMS FOR CLUBBING WITH OTHER JOURNALS.

We will cause any of the publications below to be sent together with THE BISTOURY, for one year, on the following terms:—

| | PUB. PRICE. | WITH BISTOURY. |
|---|---|---|
| American Agriculturalist | $1 50 | $1 50 |
| Scribner's Monthly | 4 00 | 4 00 |
| Peterson's Ladies' Magazine | 2 00 | 2 00 |
| Home and Health | 1 50 | 1 50 |
| Little Corporal | 1 50 | 1 50 |
| Good Health | 2 00 | 2 00 |
| Atlantic Monthly | 4 00 | 4 00 |
| Western Rural | 2 00 | 2 00 |
| Young Folks' Rural | 1 00 | 1 00 |
| Peter's Musical Monthly | 3 00 | 3 00 |
| Eclectic Magazine | 5 00 | 5 00 |
| The Galaxy | 4 00 | 4 00 |
| Hearth and Home | 4 00 | 4 00 |
| Our Own Friends | 1 50 | 1 50 |
| Godey's Lady's Book | 3 00 | 3 00 |
| Wood's Household Magazine | 1 00 | 1 00 |
| The Children's Hour | 1 25 | 1 25 |
| Saturday Evening Post | 2 50 | 2 50 |
| Phrenological Journal | 3 00 | 3 00 |
| Medical Times, Philadelphia | 4 00 | 4 08 |
| The Aldine | 5 00 | 5 00 |
| Merry's Musem | 1 50 | 1 50 |
| Ballou's Monthly | 1 50 | 1 50 |
| Arthur's Home Magazine | 2 00 | 2 00 |
| Monthly Novelet | 2 00 | 2 00 |
| Our Young Folks | 2 00 | 2 00 |
| Ohio Farmer | 2 00 | 2 00 |
| Cultivator and Country Gentleman | 2 50 | 2 50 |
| New York Independent | 2 50 | 2 50 |
| American Union | 2 50 | 2 50 |
| Rural New Yorker | 3 00 | 3 00 |
| Saturday Night | 3 00 | 3 00 |
| Ladies' Repository | 3 50 | 3 50 |
| Harper's Magazine | 4 00 | 4 00 |
| " Bazar | 4 00 | 4 00 |
| " Weekly | 4 00 | 4 00 |
| Every Saturday | 5 00 | 5 00 |
| North American Review | 6 00 | 6 00 |
| Lippincott's Magazine | 4 00 | 4 00 |
| American Farmer's Advocate | 1 00 | 1 00 |

Forward the price of any of the above publications to our address, and you will receive both journals regularly, for one year. Address

**The Bistoury**, ELMIRA, N. Y.

